# Interaction of thin polyethyleneimine layer with the iron surface and its effect on the electrochemical behavior

**DOI:** 10.1038/s41598-022-07474-z

**Published:** 2022-03-02

**Authors:** Radka Gorejová, Natália Podrojková, Katarína Sisáková, Jana Shepa, Ivan Shepa, Alexandra Kovalčíková, Ivana Šišoláková, František Kaľavský, Renáta Oriňaková

**Affiliations:** 1 Department of Physical Chemistry, Institute of Chemistry, Faculty of Science, Pavol Jozef Šafárik University in Košice, Moyzesova 11, 041 54 Košice, Slovakia; 2 Institute of Materials Research, Slovak Academy of Sciences, Watsonova 47, 040 01 Košice, Slovakia

**Keywords:** Implants, Corrosion, Biosurfaces

## Abstract

Polymer-coated metals may act as biodegradable orthopedic implants with adjustable corrosion rates. Metallic surfaces represent a dynamic system with specific interactions occurring after the material is implanted into the human body. An additional layer, in the form of polymeric thin film, changes the nature of this metal-body fluids interface. Moreover, the interaction between polymer and metal itself can differ for various systems. Iron-based material modified with a thin layer of polyethyleneimine (PEI) coating was prepared and studied as potential absorbable implant. Computational methods were employed to study the interaction between the metallic surface and polymer functional monomer units at atomic levels. Various spectroscopical and optical methods (SEM, AFM, Confocal, and Raman spectroscopy) were also used to characterize prepared material. Electrochemical measurements have been chosen to study the polymer adsorption process onto the iron surface and corrosion behavior which is greatly influenced by the PEI presence. The adsorption mechanism of PEI onto iron was proposed alongside the evaluation of Fe and Fe-PEI degradation behavior studied using the impedance method. Bonding via amino -NH_2_ group of PEI onto Fe and enhanced corrosion rate of coated samples were observed and confirmed.

## Introduction

Iron-based materials gained a noticeable amount of attention during the last years as a potential biodegradable orthopedic scaffolds^1–6^. Absorbable metallic implants can provide temporary support to the damaged tissue until their complete resorption. Different fabrication approaches have been established including casting, molding, powder metallurgy methods, arc melting, or additive manufacturing e.g.^7–15^. Emphasis is put on the preparation of biocompatible, mechanically stable load-bearing materials with appropriate degradation properties and corrosion rates matching these of human bones. Material degradation speed is a key parameter in a biodegradable implant design. The too slow corrosion rate of materials made of pure iron, therefore, represents a lively scientific issue that is being solved^12,16–20^. Several different methods used to accelerate the degradation of pure iron were introduced. Alloying with different biocompatible elements such as magnesium (Mg) or zinc (Zn)^17,21–24^, or controllable changes in material porosity were already studied^22,25,26^. The desired acceleration of corrosion can be achieved by using these methods, however, the use of elements that are non-toxic and choosing a degree of porosity that does not negatively affect the resulting mechanical properties of the material is still challenging. Another way to achieve enhanced corrosion speed of iron is to apply a thin polymeric layer on its surface. Several studies dealing with this problem, where poly(lactic-co-glycolic) acid (PLGA)^27^ or polyethyleneglycol (PEG)^28–30^ has successfully proven the positive effect of the polymer used not only in the terms of degradation acceleration but also in enhancing biological and mechanical properties. In our previous studies^31,32^, polyethyleneimine (PEI) has been chosen due to its non-harmful biological properties and corrosion-enhancing potential. PEI is a polycationic polymer that can serve as a drug or gene carrier and has been also used in cell attachment, DNA isolation, or wastewater problem studies^33–36^. We have found that PEI can affect the corrosion speed of pure iron in a positive way (to accelerate it) and a corrosion mechanism of degradation of Fe-PEI material in simulated body fluids was proposed^32^. Since this polymer seems to be a very promising candidate in a biodegradable scaffold design, there is a need to examine its interaction with a metallic substrate in detail. Not only the interaction of the metal–polymer system but also its influence on the corrosion process were studied in this paper by molecular modeling and electrochemical methods. Gained information can bring a broader knowledge about the system which is crucial for the design of safe biomaterial intended for long-term use in the human body. PEI-coated materials represent an original concept with a high chance to play an important role in biodegradable implant development. Moreover, the flexibility of coating procedures with the possibility to easily scale the coating parameters and therefore to model ideal corrosion properties can adapt to the needs of medical practice.

## Experimental

### Sample preparation

The spherical iron powder obtained from Alfa Aesar (Germany) was used to fabricate iron pellets (Ø 10 mm, h = 2 mm). Powder with a purity of 99.5% was cold pressed into cylinders at the pressure of 600 MPa and subsequently sintered in a tube furnace in the reductive atmosphere composed of 10% H_2_ and 90% N_2_. Sintering was performed for 1 h at 1120 °C. Polyethyleneimine (PEI, branched, 60–100 kDa, 50% (w/v) in H_2_O, Sigma Aldrich, USA) was diluted to the concentration of 5 wt% in 96 vol.% ethanol (Centralchem, Slovakia). Iron pellets were ground with SiC papers (240, 800, and 1500 grits) and UV-cleaned in acetone and ethanol, each for 10 min before the polymer coating application. The coating was applied by the dip-coating technique. The ground and cleaned pellets were immersed in the PEI solution for 90 min and dried at 37 °C for 12 h in the drying chamber to evaporate the solvent and obtain a thin PEI layer.

### Sample surface characterization

The scanning electron microscope (SEM) (JEOL JSM-7000F, Japan with EDX INCA and ZEISS AURIGA COMPACT) was employed to examine the surface morphology of pure Fe and PEI coated iron samples.

Atomic force microscopy (AFM) measurements were performed by the Veeco/Bruker Dimension Icon microscope in the tapping mode. This method allowed to obtain high-definition data of surface topology in both height and phase contrast modes. Images were processed in NanoScope Analysis and Gwyddion software.

Macroscopic photos were made by Carl Zeiss Stemi 2000-C stereomicroscope equipped with Zeiss AxioCam ICc 5 cameras.

Confocal microscopy was performed by the Plu Neox 3D optical surface profiler by SENSOFAR with 20 × objective. The size of the single image was 637 × 478 µm (approx. 637 × 478 µm). Raman spectra were recorded by XploRA ONE Raman microscope from Horiba Jobin Yvon with laser wavelength of 532 nm and magnification objective—10 × in range of 100–4000 cm^-1^.

### Computational methods

All DFT calculations were performed using Grid-Based Projector-Augmented Wave GPAW code in Atomic Simulation Environment (ASE) software package^37^. To describe electronic interactions of the modes revised Perdew-Burke-Erzerhof (rPBE) which uses double numerical plus polarization (dnp) was applied with Generalized Gradient Approximation (GGA) and FD modes. The adsorption of ethylamine on the iron surface was studied considering the long-range dispersion forces, to correctly describe the interaction between the ethylamine molecule and the iron surface, therefore Tkatchenko-Scheffler correction for long-range interactions was applied. The surface of Fe(110) was built from the Fe unit cell according to the crystallographic data of pure Fe applied in experimentalresearch (not presented in this manuscript) and optimized for further computational studies of Fe and ethylamine interactions. The energy minimization of the unit cell was accomplished when the unit cell achieved full relaxation and the forces were smaller than 0.05 eV/Å. The Fe bcc (110) surface was built from an optimized unit cell and consisted of 36 Fe atoms, which were arranged in 4 layers (3 × 3 × 4). To ensure the complete disappearance of the wave function at the edges of the cell, the vacuum was set to 10 Å on both sides. The lower 2 atomic layers were fixed which allowed the relaxation of the two atomic layers on the top. When optimizing the structure, the symmetry constraints were turned off so that the iron atoms could move in all directions, reorient, and thus find their structure with minimal adsorption energy. For the interactions between adsorbed ethylamine molecule and Fe surface, the two layers of the iron surface were allowed to relax indefinitely until the residual forces at all atoms reached less than 0.05 eV/Å. The adsorption energy of adsorbed structures on the Fe surface was calculated according to Eq. 1

1 $${E}_{ads}={E}_{S+M}-({E}_{S}+{E}_{M})$$

where *E*_*ads*_ is the adsorption energy of ethylamine on Fe surface, *E*_*S*+*M*_ is the total energy of the adsorbate–substrate system; *E*_*S*_ is the energy of an uncovered surface, and *E*_*M*_ is the energy of the isolated ethylamine molecule.

### Electrochemical testing

Electrochemical impedance spectroscopy (EIS) measurements were carried out in the frequency range from 10 MHz to 100 kHz with an AC perturbation amplitude of 10 mV and DC potential equal to the open circuit potential (OCP) determined for 60 s before the measurements. The electrochemical tests were conducted using Multichannel Autolab M204 potentiostat (Metrohm AG, Herisau, Switzerland) in 5 wt.% PEI solution for polymer adsorption kinetic study (for 90 min) and in Hanks´ solution for the study of corrosion behavior (before and after 60 min of immersion). A three-electrode system with Ag/AgCl/KCl (3 mol/l) as a reference electrode, platinum counter electrode, and uncoated or PEI coated iron sample as the working electrode was used.

## Results

### Adsorption energy determined by DFT calculations

**Figure 1:**
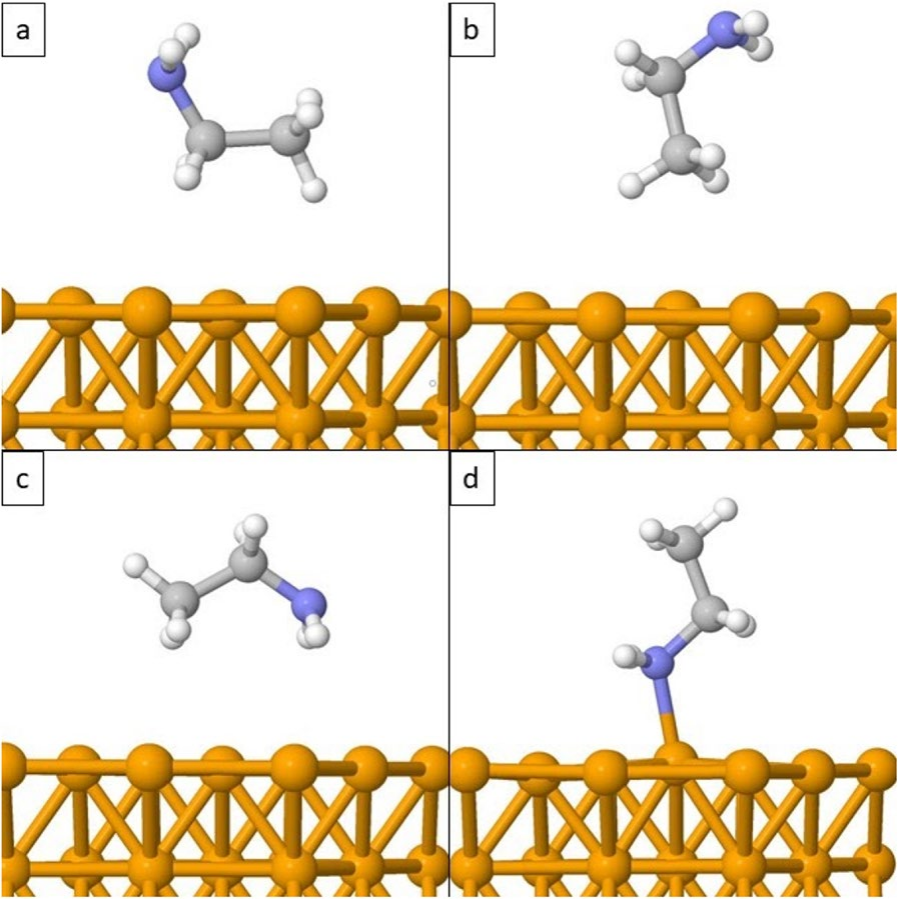
Optimization of an ethylamine molecule turned in different directions towards the surface of Fe (110): linear positioning of CH_3_CH_2_- towards Fe(110) (**a**), vertical positioning of CH_3_CH_2_-towards Fe(110) (**b**), linear positioning of CH_3_CH_2_NH_2_ molecule towards Fe(110) (**c**) and vertical positioning of ethylamine molecule with -NH_2_ end adsorbed to Fe(110) (**d**).

The ethylamine molecule was selected as a monomer unit of polyethyleneimine (PEI) to study the method of PEI bonding to the Fe(110) surface. To determine the most stable adsorbed ethylamine molecule on the Fe(110) surface and adsorption energy of such system, several structures of ethylamine differently oriented towards Fe surface have been included for optimization. Figure 1 displays optimized structures of C_2_H_5_NH_2_ and Fe(110). Four possible rotations of the ethylamine molecule to the surface were considered. In the first case, the ethylamine molecule was placed with a carbon chain linearly towards Fe(110) surface (Fig. 1a). In the second case, ethylamine was turned vertically with a carbon chain oriented towards Fe(110) (see Fig. 1b). Figure 1c shows that the whole ethylamine molecule was placed linearly to the surface and Fig. 1d presents the position of the ethylamine molecule vertically towards the Fe(110) surface, with the terminal -NH_2_ group facing the surface (Fig. 1d). As it can be seen, ethylamine molecule prefers to bond to Fe(110) surface through -NH_2_ group.

**Figure 2:**
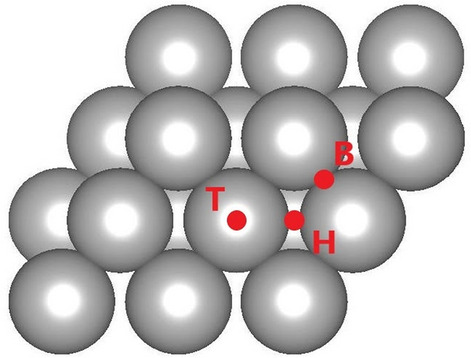
Locations on the Fe(110) bcc surface of the presumed adsorption of the ethylamine molecule on which the molecule was placed before optimization: at the top of the Fe-top atom (T), in the gap between the Fe-hollow (H) atoms, and at the bridge between the Fe-bridge atoms (B).

When the most stable and most probable bonding of C_2_H_5_NH_2_ with Fe(110) was found, different locations of ethylamine molecules adsorbed on the Fe surface through the -NH_2_ group were computationally examined. To determine the site of adsorption on the bcc Fe(110) surface, the ethylamine molecule was placed at three sites. Figure 2 shows sites on the surface of bcc Fe(110), where the adsorption of the ethylamine molecule on the surface most probably occurred: (1) the top position (T), where the ethylamine molecule was placed on the surface of the iron atom (Fig. 2—marked as a T position), (2) the bridge position (B), where the ethylamine molecule was in the space between two iron atoms (Fig. 2—marked as a B position) and (3), the hollow position (H), where the ethylamine molecule was in the interatomic gap formed between the four iron atoms and Fe atom located below these atoms covering the gap from beneath (Fig. 2—marked as an H position).

**Figure 3:**
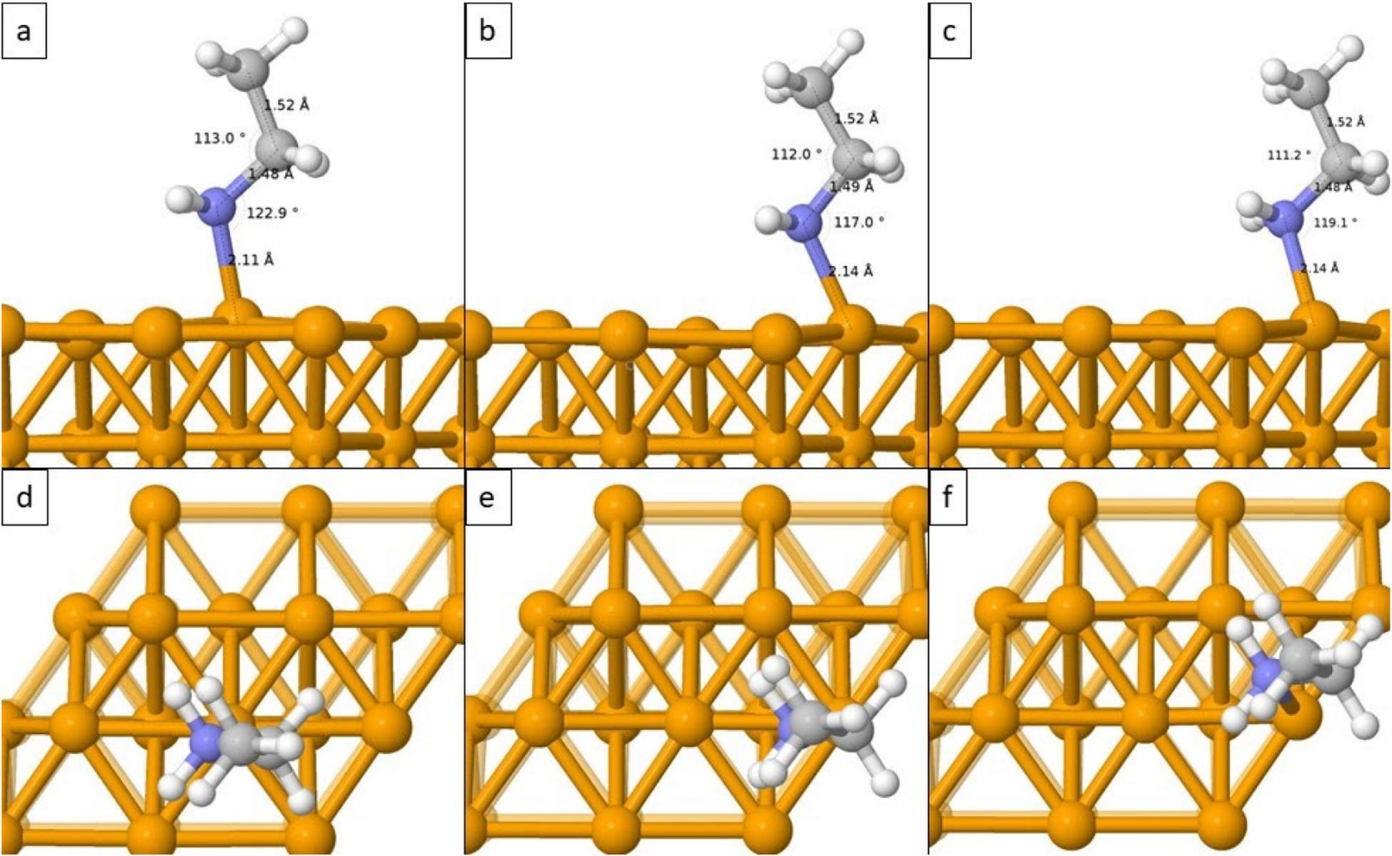
The adsorption of ethylamine molecule on the surface of Fe(110) displayed from the front top (**a**), hollow (**b**), bridge (**c**) and from the above top (**d**), hollow (**e**), bridge (**f**).

The results of adsorption energy showed that adsorption of ethylamine molecules is possible on all sites. Table 1 summarizes the adsorption energies achieved by each system during optimization and Fig. 3 presents optimized adsorbed C_2_H_5_NH_2_ structures on different sites of Fe(110) surface. C_2_H_5_NH_2_ adsorbed on T site resulted in adsorption energy − 1.38 eV, the adsorption energy of C_2_H_5_NH_2_ adsorbed on H site was − 1.21 eV and C_2_H_5_NH_2_ adsorbed on B site resulted in adsorption energy − 1.25 eV (Table 1). The adsorption energies show that in all cases the binding of ethylamine molecule to Fe(110) is stable and the adsorption energy is not varying highly.

**Table 1:** Summary of adsorption energies in eV and kJ/mol, bond lengths, and angle sizes of optimized adsorbed ethylamine molecules in the top, hollow, and bridge positions of the Fe (110) surface.

Site	Adsorption energy (eV)	Adsorption energy (kJ/mol)	Bond lengths (Å)	Angle sizes (°)
Top (T)	− 1.38	− 133.15	d_(Fe–N)_ = 2.11	(Fe–N–C_1_) = 122.9
			d_(N–C1)_ = 1.48	(N–C_1_–C_2_) = 113.0
			d_(C1–C2)_ = 1.52	
Hollow (H)	− 1.21	− 116.81	d_(Fe–N)_ = 2.14	(Fe–N–C_1_) = 117.0
			d_(N–C1)_ = 1.49	(N–C_1_–C_2_) = 112.0
			d_(C1–C2)_ = 1.52	
Bridge (B)	− 1.25	− 120.73	d_(Fe–N)_ = 2.14	(Fe–N–C_1_) = 119.1
			d_(N–C1)_ = 1.48	(N–C_1_–C_2_) = 111.2
			d_(C1–C2)_ = 1.52

Figure 3 displays how the ethylamine molecule binds to the surface of Fe(110) when placed on different sites. The nitrogen (N) atom prefers the adsorption on the T site since the optimized structures show the movement of the N atom from H (Fig. 3b, e) and B position (Fig. 3c, f) towards the T site, although the reposition is not complete. The atom bonds and angles also change slightly providing very similar structures in all cases. Therefore, the results demonstrate that the most possible adsorption of C_2_H_5_NH_2_ on the Fe(110) surface occurs on the T site with an adsorption energy of − 1.38 eV and with a Fe–N bond of 2.11 Å.

### Surface morphology before corrosion

**Figure 4:**
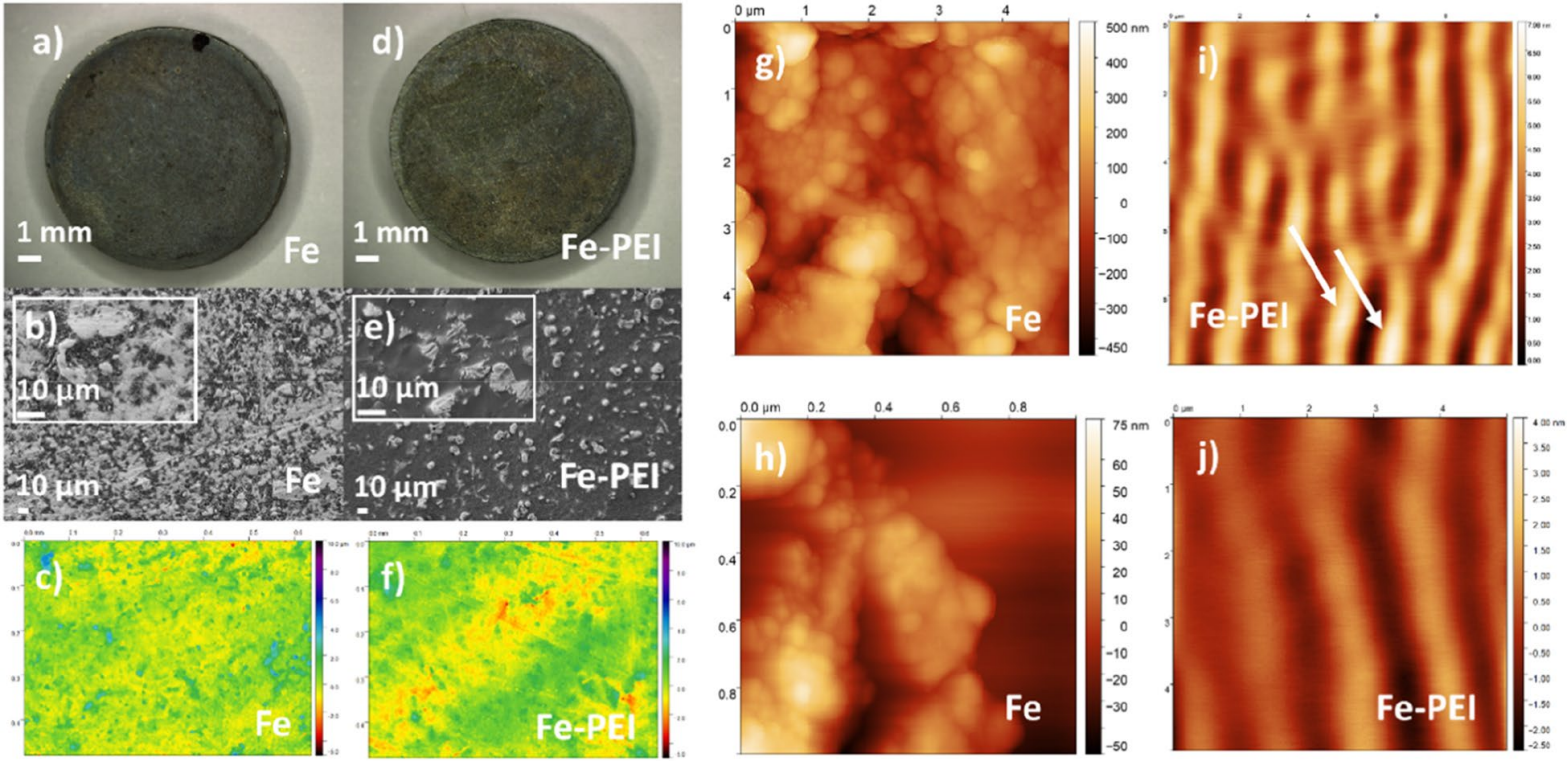
The differences between the surface morphology of pure iron (**a**–**c**, **g**,**h**) and polymer (PEI) coated samples (**d**–**f**, **i**, **j**) Macroscopic photos (**a**,**b**) along with SEM (**b**,**e**), confocal microscopy (**c**,**f**) and AFM (**g**–**j**) were taken to compare the effect of the thin layer application.

To record the topology changes after coating of bare metals and after corrosion occurred, confocal and optical microscopy were used along with the SEM and AFM (which were used for detailed scans). The surface morphology of pure iron and PEI coated samples were studied and compared. Figure 4 depicts the macroscopic and microscopic photos of the analyzed samples. The surface of the bare iron pellets and polymer-coated samples were studied using the SEM method to visualize and evaluate polymer distribution on the specimen surface. A comparison of the surface of pure iron and PEI coated samples is depicted in Fig. 4. In the case of pure iron, an uneven, bumpy surface can be observed (Fig. 4a, b) which is characterized by irregular protrusions of various sizes. Scratches present on the surface were caused by the mechanical treatment of the samples. After the coating, small irregularities were filled with a thin layer of polyethyleneimine (Fig. 4d, e), while the higher-height protrusions remained uncovered (Fig. 4e, i). Confocal microscopy micrographs of non-corroded samples are depicted in Fig. 4c, f with only slight differences for coated and uncoated samples, which are more evident from the results of SEM and AFM analyses.

To study the microscopic structure of prepared material, which is outside the imaging capability of previous methods, the AFM method has been chosen. This method allows the detection of the sub-grain structure of the sintered material. Only samples of pure iron before and after corrosion and Fe-PEI sample before corrosion were scanned due to the too high roughness of the Fe-PEI sample after corrosion. Scans obtained from AFM measurements are depicted in Fig. 4g–j. The structure of pure iron (Fig. 4g, h) before corrosion with typical globular clusters was identified. The average size of spherical iron subgrains was ranging around 50 nm. On the other hand, the microstructure of polymer-coated samples significantly differed from the samples of pure iron. PEI covered spherical iron particles and formed longitudinally arranged polymer waves (Fig. 4i, j) which were concentrated mainly in the material valleys. White spots on the sample surface (Fig. 4i, white arrows) represent uncoated iron bumps.

### Impedance measurements

#### Polymer adsorption

**Figure 5:**
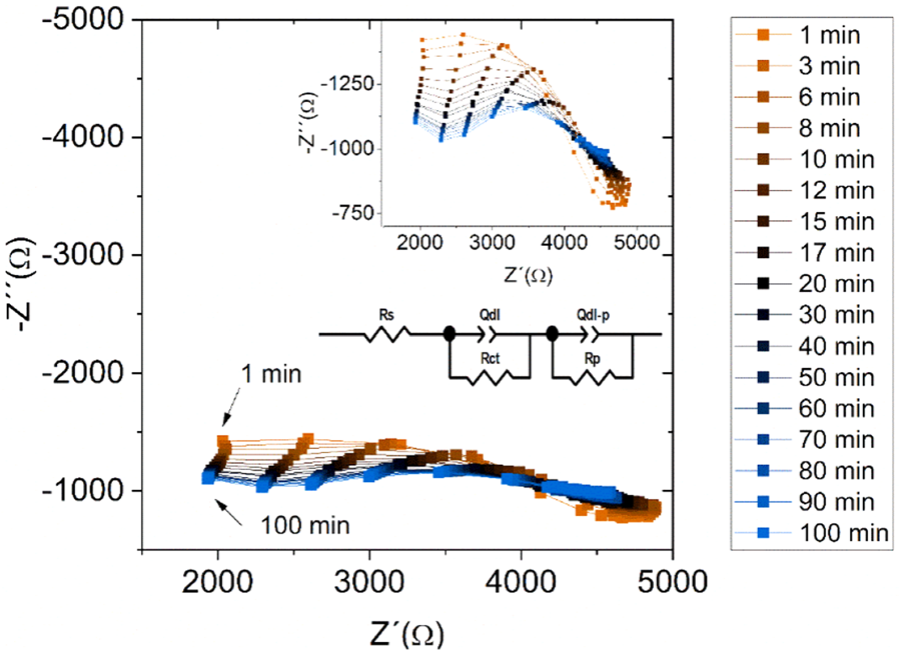
Impedance spectra for Fe sample immersed in the 5 wt.% PEI solution for 100 min to simulate the dip-coating process.

Polymer adsorption to iron surface during 90 min of the dip-coating process was studied by the electrochemical impedance spectroscopy method. EIS spectra were measured at various time points during the coating process (Fig. 5) and were fitted using the equivalent circuit shown in the same figure.

The equivalent circuit is formed from the next electrical elements: *R*_*s*_ is solution resistance, *Q*_*dl*_ is constant phase element corresponding to double layer, *R*_*ct*_ is charge transfer resistance, *Q*_*dl-p*_ is constant phase element corresponding to the polymer film, and *R*_*p*_ represents polymer film resistance. *Q*_*dl*_ was calculated via the following equation (Eq. 2)

2 $${Q}_{dl}= {\left(2\pi {f}_{max}{R}_{ct}\right)}^{-1}$$

where *f*_*max*_ is the frequency corresponding to the maximum of the imaginary impedance component.

It was expected that the Fe-PEI bond formation influences the values of electrical elements. After only 3 min, one flattened semicircle could be observed, which diameter decreases. After 10 min, an indication of a second semicircle could be observed. According to literature, high-frequency semicircle corresponds to the properties of the organic film in the organic/metal system. On the other hand, low-frequency semicircle provides information about the reaction of the metal component^38^. So, the formation of the second semicircle in the high-frequency region could exactly confirm the organic film formation on the Fe surface. Moreover, PEI is a favorable molecule for complexing heavy metal ions based on the literature^36^.

#### Electrochemical corrosion behavior

The EIS measurement was performed with the pure iron pellet and PEI-coated pellet immersed in Hanks’ solution. The EIS spectra of all samples were fitted using the same equivalent circuit as in the previous case.

**Figure 6:**
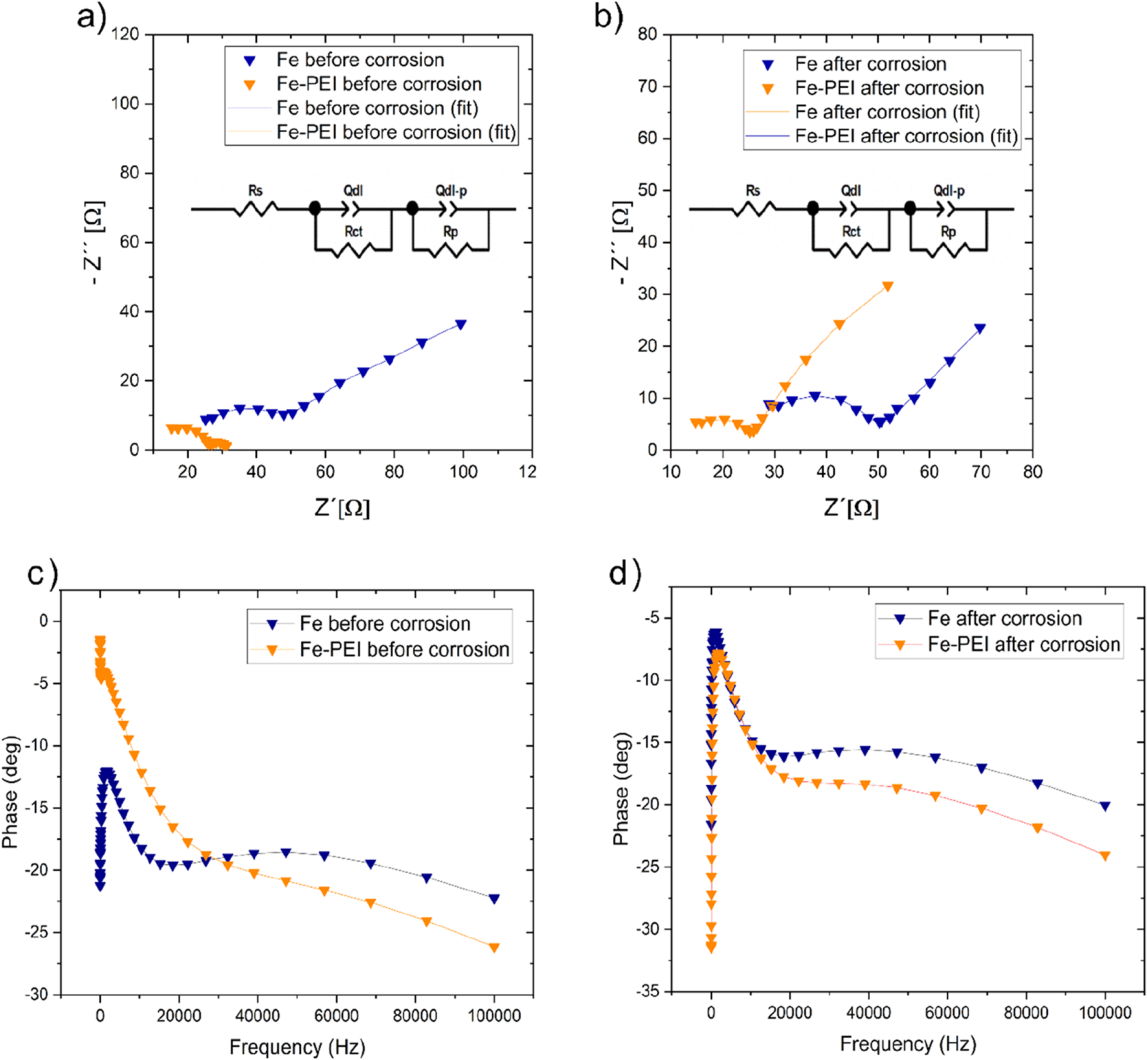
Nyquist (**a**,**b**) and Bode (**c**,**d**) plots of samples (Fe, Fe-PEI) before and after 60 min of corrosion.

The Bode diagrams (Fig. 6c, d) exhibits two peaks of phase lag at high and low frequencies for both samples with and without PEI. So, the time constant observed in the high- and low-frequency region is related to corrosion product film or PEI film and the charge transfer process of the corrosion reaction, respectively.

### Degradation behavior and products

**Figure 7:**
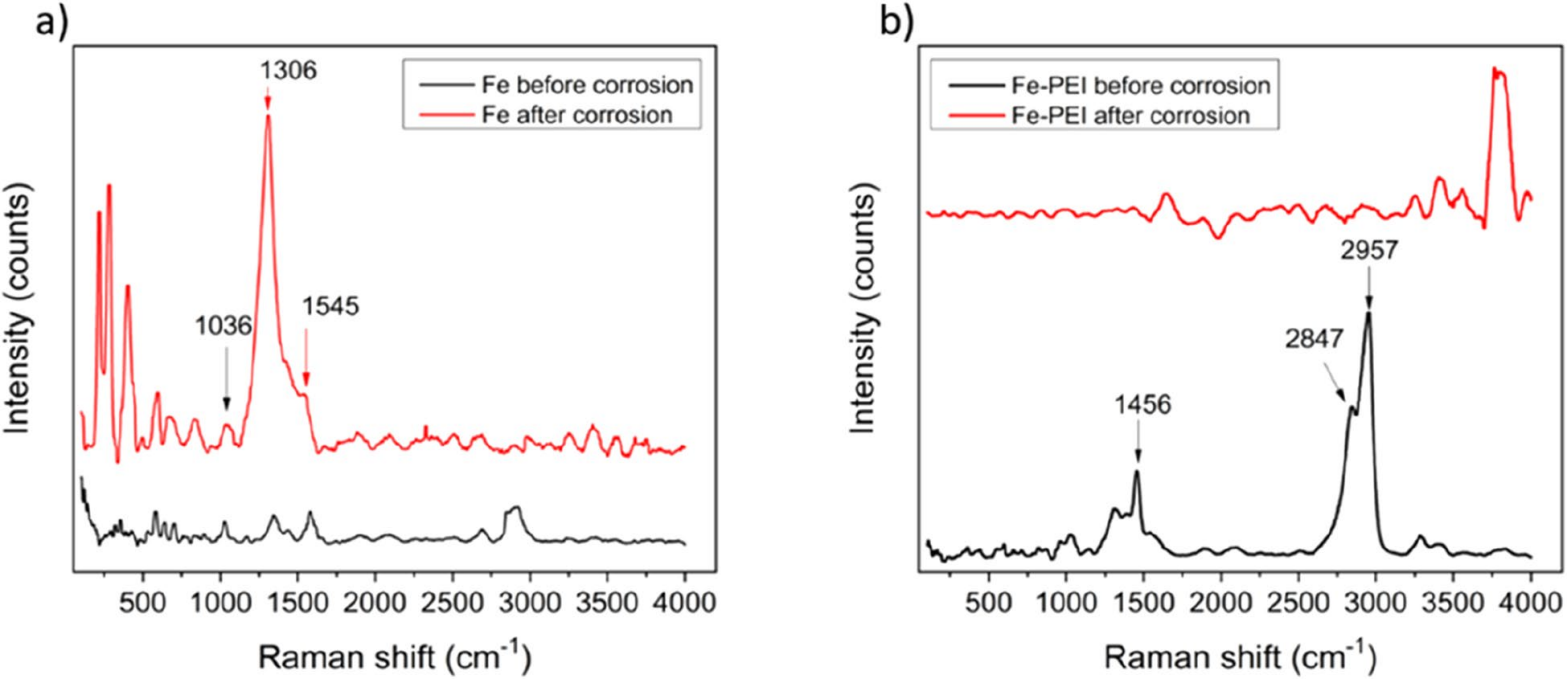
Raman spectra of Fe (**a**) and Fe-PEI (**b**) samples series before and after 60 min of corrosion in simulated body fluids.

The degradation process takes place during 60 min of immersion in Hanks’ solution. Raman spectra recorded for both PEI modified and pure Fe sample series are shown in Fig. 7. Spectra for Fe shows the presence of a thin oxide layer on the surface, which partially acts like the passivation layer. As can be seen from the spectra, there is no evidence of the deposits on the corrosion products for the pure Fe series. Peaks visible for the Fe sample after corrosion mostly refer to the components of the Hanks´ solution. Regarding the series of Fe-PEI: on the first spectrum characteristic for PEI, nice and sharp peaks at 1456, 2847 and 2957 cm^−1^ are visible. Other spectra show the presence of corrosion reaction deposits and similarly to the pure Fe samples—components of the Hank solution on the surface. The absence of the PEI on the treated surfaces refers to the dissolution of the layer involved in the corrosion process.

## Discussion

Optical, confocal, SEM, and AFM methods confirmed the successful coating of PEI on the surface of the iron pellets. DFT calculations and EIS measurements were further employed to study the adsorption process mechanism in detail. From the adsorption energies and calculations, the ethylamine molecule was adsorbed solely by turning the terminal -NH_2_ group towards the iron surface. Since the ethylamine is a simple aliphatic amine with a free electron pair on the nitrogen, it can act as a nucleophilic agent and at the same time form a coordination bond. PEI chelating ability is well known and different polymer structures (linear vs. branched) and parameters (pH, viscosity e.g.) were studied and compared^39,40^. Since linear PEI has only secondary amino groups in the main chain, branched PEI has primary, secondary, and tertiary amino groups accessible thus stronger metal-binding properties^41^. Kislenko et al.^39^ studied and confirmed complex formation between PEI molecule and metal (copper, nickel, cobalt) ions. They found the reaction of complex formation to be controlled by the metal ions diffusion into the polymer globule in solution which may also apply to iron studied in our work. Also, alkali or alkaline earth metals (present in the Hanks´ solution) are not chelated by PEI at any pH^41^ which favors the Fe-PEI chelation over others and may contribute to the more rapid degradation rate. We propose the following mechanism of degradation of Fe-PEI samples along with the Fe-PEI complexation reactions, where *R* refers to the H(NHCH_2_CH_2_)_n_NH_2_ (*n* differs regarding the molecular weight (*M*_*w*_) of branched PEI. For example, for PEI of *M*_*w*_ = 1800; *M*_*w*_ = 70,000 or *M*_*w*_ 750,000 is *n* ~ 42, ~ 1628 and ~ 17,442 respectively^42^):

3 $$Fe+R-N{H}_{2}\to FeR-N{H}_{ad}+{H}^{+}+{e}^{-}$$

4 $$Fe+R-N{H}^{-}\to FeR-N{H}_{ad}+{e}^{-}$$

5 $$FeR-N{H}_{ad}\to FeR-N{H}^{+}+ {e}^{-}$$

6 $$FeR-N{H}^{+}+ {H}^{+}\to F{e}^{2+}+R-N{H}_{2}$$

It is important to note that due to the complex structure of the branched polymer, this model is simplified.

Also, primary, secondary, and tertiary amines oxidation occurs. Generally, branched PEI (BPEI) is more prone to oxidation than linear PEI (LPEI)^43^. Primary amines will react first due to their higher reactivity. Typical oxidation products of primary, secondary and tertiary amines are amphoteric hydroxylamines (–NH–OH)/nitroso species (–N = O)/nitro species (–NO_2_), secondary hydroxylamines (RR′–N–OH), and tertiary N-oxide [R_3_N(+)–O(–)], respectively. A decrease in the number of PEI reactive primary amines may also significantly lower the material toxicity^44^.

**Figure 8:**
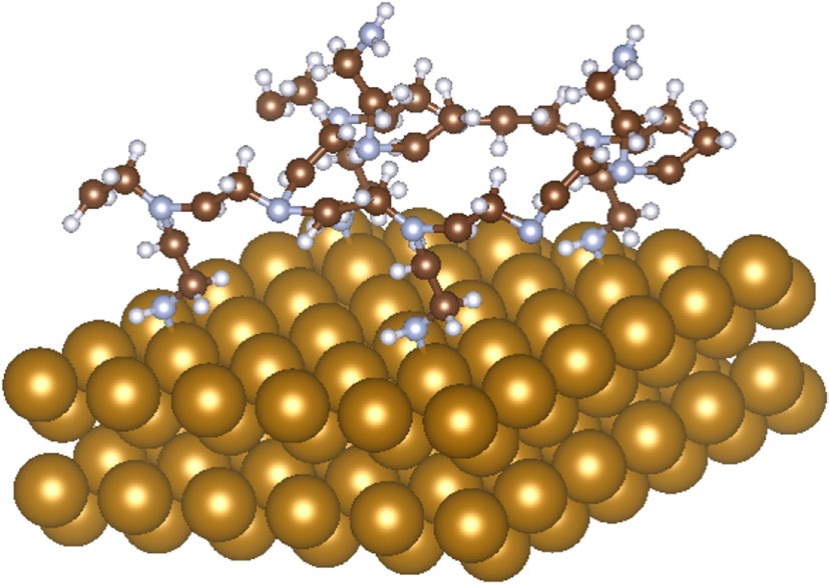
Conceptual representation of polyethyleneimine (PEI) bound to the iron surface through -NH_2_ group.

Despite the different rotation of the molecule and its location at different surface sites, the molecule bound in each case directly to the iron atom via the -NH_2_ group, which is consistent with previous experimental FT-IR results^31^ which also indicated the binding of the molecule to the iron surface via the -NH_2_ group. In the case of placing the ethylamine molecule in hollow or bridge positions, the molecule draws away from the iron surface. The lowest value of adsorption energy was achieved with a surface in which the molecule was placed directly above the Fe(110) atom with a value of -1.38 eV. The Fe–N bond length, in this case, was d_(Fe–N)_ = 2.11 Å. Schneider et al. studied iron complexes^45^ and found, that in the case of high-spin (HS) Fe^II^, the average Fe–N bond distances were d_(Fe–N)_ = 2.19 Å, while in the low-spin (LS) Fe^II^ state the average d_(Fe–N)_ = 1.97 Å. Since the modeled data dealt only with the ideal atomic iron surface due to the real system complexity, oxidized iron species are expected to be present on the real specimen. Zheng et al.^46^ focused on how the spin state affects the iron-binding sites in macromolecular structures. They discerned two populations of Fe^II^ binding sites which corresponded to Fe–N R_0_ parameters of 1.57 Å for LS iron and 1.76 Å for HS iron. A similar distribution was observed for Fe^III^ as well. Based on results from computational modeling of monomer unit of PEI, the potential structure of branched polymer layer adsorbed on Fe(110) surface was designed (Fig. 8).

The fitted Nyquist plots of samples before and after corrosion are shown in Fig. 6. The equivalent circuit was chosen because of agreement with experimental results for all samples. To characterize the solid/solution interface, the *R*_*ct*_ and *Q*_*dl*_ were studied. The *R*_*ct*_ value increase after the corrosion for all samples. The resistance increase relates to the formation of corrosion products. In the case of Fe-PEI, the double-layer capacitance decreases which indicated the enhanced formation of degradation products. According to the results for the Fe-PEI sample, also the capacitance decreased because of the formation of corrosion products and surface degradation could be expected. Both semi-circles in the high and low frequencies correspond to the capacitive behavior. Nyquist diagrams for Fe samples display two semicircles, which correspond to the contact resistance of metal at high-frequency and the corrosion reaction of the iron sample at low-frequency region. In the case of metal-polymer samples, interpretation of EIS spectra was discussed above. Impedance spectra were fitted by using *ZView* software and the values appropriate for all samples before and after the corrosion process are listed in Table 2. In both cases (before and after corrosion), samples coated with PEI display almost two times lower values of resistance. The charge transfer resistance decreases from 13.9 to 8.25 Ω and from 19.04 to 10.13 Ω for samples before and after corrosion, respectively. The diameter of both semicircles is lower in the case of Fe-PEI samples in comparison with Fe samples. This behavior indicates easier charge transfer, which insinuates lower stability of Fe-PEI samples, therefore their easier corrosion. Moreover, the constant phase element values increase in both cases before and after corrosion. The value increment was higher for samples before corrosion (from 0.70 to 2.82 µF).

**Table 2:** Values of impedance parameters for Fe and Fe-PEI samples before and after corrosion.

Impedance parameter	Fe		Fe-PEI	
	Before corrosion	After corrosion	Before corrosion	After corrosion
*R*_*ct*_ (Ω)	13.9	19.04	8.25	10.13
*Q*_*dl*_ (µF)	0.70	1.28	2.82	1.44

Higher degradation speed resulting in more rapid corrosion product formation represented by the higher resistance value in EIS measurements was observed for Fe-PEI samples indicating their higher corrosion speed when compared to the pure iron. The size and steric effect of the used coating units may also influence their performance either as corrosion inhibitors or catalysts. If the nanosized coatings are used, the particles are blocking the corrosive elements from diffusing into the surface of the substrate^47^. Due to their size and high density on the material surface, these may act as a good corrosion inhibitor. On the other hand, homogeneous access of corrosive species from the solution to the fresh metallic surface needs to be ensured if corrosion enhancement is wanted. Therefore, no steric hindrance should appear either during corrosion products formation or the penetration of corrosive solution to the surface.

Raman spectroscopy studies were employed to evaluate the composition of sample surface before and after corrosion occurred. Visible peaks corresponding to the polyethyleneimine coating were present for Fe-PEI samples before corrosion which confirms the successful coating process. Moreover, the intensity of peaks corresponding to pure PEI decreased after corrosion suggesting that polymer dissolution in simulated body fluids occurred as soon as after 60 min. This finding is of great importance for designing the implant with the non-toxic polymer concentration which may be released over a very short period after implantation. On the other hand, quick release of the drugs incorporated into such a layer may be beneficial in the acute phase after surgery with the higher risk of adjacent tissue inflammation. The composition of deposited degradation products did not significantly differ for Fe and Fe-PEI samples, however, their morphology did. General iron corrosion mechanism starts with material oxidation reactions (Eqs. 7 and 8)

7 $${\text{Fe}}{\to {\text{Fe}}}^{2+}+ \text{ 2} {\mathrm{e}}^{-}\mathrm{ Anodic}$$

8 $${\mathrm{O}}_{2}+2 {\mathrm{H}}_{2}\mathrm{O}+4 {\mathrm{e}}^{-}\to 4 {\mathrm{OH}}^{-}\mathrm{ Cathodic}$$

followed by the creation of iron (Fe^2+^ and Fe^3+^) hydroxides (Eqs. 9–11) and magnetite (Eq. 12)^48,49^.

9 $${\mathrm{Fe}}^{2+}+{2\mathrm{ OH}}^{-}\to {\mathrm{Fe}(\mathrm{OH})}_{2}$$

10 $${\mathrm{Fe}}^{2+} \to {\mathrm{Fe}}^{3+}+{\mathrm{e}}^{-}\mathrm{ In \,alkaline \,solutions \,and \,presence \,of \,oxygen}$$

11 $${\mathrm{Fe}}^{3+}+{3\mathrm{ OH}}^{-}\to {\mathrm{Fe}(\mathrm{OH})}_{3}$$

12 $${\mathrm{Fe}(\mathrm{OH})}_{2}+2\mathrm{ FeO}(\mathrm{OH})\to {\mathrm{Fe}}_{3}{\mathrm{O}}_{4}+{\mathrm{H}}_{2}\mathrm{O\, In \,presence\, of\, oxygen \,and \,Cl}^{-} \mathrm{ ions}$$

During immersion in Hanks´ solution, the following reactions (Eqs. 13–20) take place according to Zhang^50^:

13 $${\mathrm{Fe}(\mathrm{OH})}_{2}+{\mathrm{Cl}}^{-}\to \mathrm{FeClOH}+{\mathrm{OH}}^{-}$$

14 $$\mathrm{FeClOH}+{\mathrm{H}}^{+}\to {\mathrm{Fe}}^{2+}+{\mathrm{Cl}}^{-}+{\mathrm{H}}_{2}\mathrm{O}$$

15 $${\mathrm{Fe}(\mathrm{OH})}_{3}+{2\mathrm{ Cl}}^{-}\to {\mathrm{FeCl}}_{2}\mathrm{OH}+{2\mathrm{ OH}}^{-}$$

16 $${\mathrm{FeCl}}_{2}\mathrm{OH}+{\mathrm{H}}^{+}\to {\mathrm{Fe}}^{3+}+{2\mathrm{ Cl}}^{-}+{\mathrm{H}}_{2}\mathrm{O}$$

17 $${2\mathrm{ PO}}_{4}^{3-}+{3\mathrm{ Ca}}^{2+}\to {\mathrm{Ca}}_{3}{(\mathrm{PO}}_{4}{)}_{2}\downarrow$$

18 $${2\mathrm{ PO}}_{4}^{3-}+{3\mathrm{ Mg}}^{2+}\to {\mathrm{Mg}}_{3}{(\mathrm{PO}}_{4}{)}_{2}\downarrow$$

19 $${2\mathrm{ PO}}_{4}^{3-}+{3\mathrm{ Fe}}^{2+}+{8\mathrm{ H}}_{2}\mathrm{O}\to {\mathrm{Fe}}_{3}{(\mathrm{PO}}_{4}{)}_{2}.{8\mathrm{ H}}_{2}\mathrm{O}$$

20 $${\mathrm{PO}}_{4}^{3-}+{\mathrm{Fe}}^{3+}\to {\mathrm{FePO}}_{4}\downarrow$$

**Figure 9:**
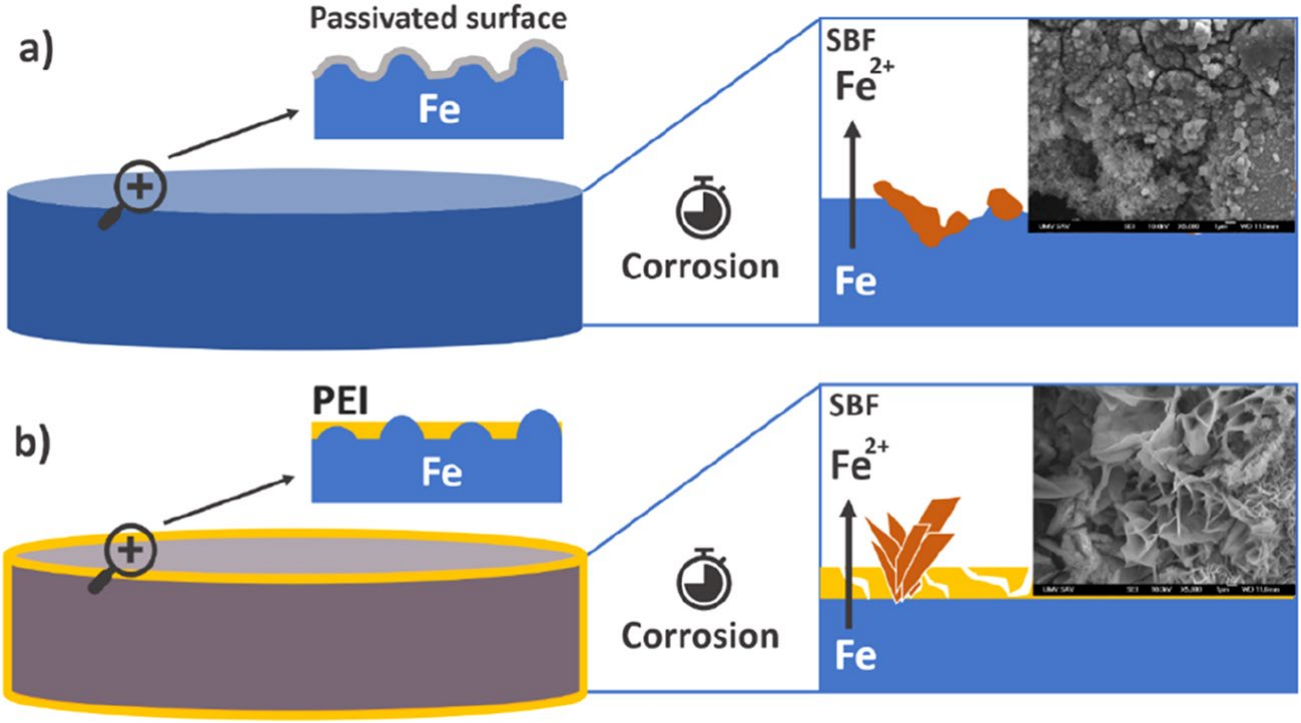
Scheme of degradation process ongoing on the surface of pure Fe pellet (**a**) compared to process ongoing on the PEI-coated material (**b**).

In the case of pure iron, intensive cracking of surface along with the typical pitting and rather spherical corrosion products were present while in the case of Fe-PEI, more crystal-like products were found (Fig. 9), composed of different chemical components described in Eqs. 10–17. This is due to the different corrosion mechanisms of these samples, which were described in detail by Oriňaková et al.^32^.

The iron-based material corrosion usually starts with the oxohydroxide ferrihydrite (Fe(III)) precipitation during the hydrolysis. However, this form is thermodynamically unstable and the creation of more crystalline oxohydrides is favored. Ferrihydrite is subsequently turned into lepidocrocite, goethite, and magnetite^51,52^. Secondary mineral transformation rates are determined by the uptake of electrons from solution (via dissolved Fe(II)) and also, chloride ions influence the formation of flaked rust^53^. Lepidocrocite production will be highest at relatively low surface-associated Fe(II) concentrations, with these conditions more likely at lower pH^53^. Based on the results obtained from the SEM analysis, we assume that the production of lepidocrocite is favored on the Fe-PEI surface while the goethite represents the prevailing form in the case of pure iron. The polycationic character of the polymeric layer (which attracts negatively charged chloride ions) along with the changes in pH during its dissolution in simulated body fluids may be responsible for the favored lepidocrocite production on Fe-PEI samples.

## Conclusions

Iron-based biomaterials represent a promising candidate as biodegradable orthopedic scaffolds. Coating with polymeric layers is an effective way of treating the problem of their insufficient corrosion speed. Knowledge of the polymer and metal interaction is therefore crucial for a complex understanding of their behavior in the human body. Iron pellets were prepared by powder metallurgy and coated with a thin polyethyleneimine layer which has already proven its beneficial effect on enhancing corrosion speed. The polymer adsorption process on the metallic surface was studied in detail using molecular calculations. The results of DFT simulations clearly showed that the ethylamine molecule is adsorbed on the surface of bcc Fe (110). Despite the different rotation of the molecule and its location at different surface sites, the molecule is bound in each case directly to the iron atom via the NH_2_ group, which is consistent with experimental results which also indicated the binding of the molecule to the iron surface via the -NH_2_ group. In the case of placing the ethylamine molecule in hollow or bridge positions, the molecule moved away from the Fe surface. The lowest value of adsorption energy and the most stable system was achieved with a surface in which the molecule was placed directly above the Fe(110) atom with a value of adsorption energy -1.38 eV. The coordination character of the iron-organic coating layer is expected to take a place. Electrochemical measurements confirmed the successful polymer adsorption and enhanced corrosion speed of PEI-coated iron samples. The double-layer capacitance of Fe-PEI samples decreased after corrosion, which indicated the enhanced formation of degradation products, while an increase was detected for pure Fe. Detailed characterization of the Fe-PEI system therefore should help in the understanding of various polymer-coated degradable metals behavior. However, the biological performance of such material remains the most crucial and needs to be addressed soon.

## Data Availability

Data available on request from the author.
